# Long-term safety and efficacy of givinostat in polycythemia vera: 4-year mean follow up of three phase 1/2 studies and a compassionate use program

**DOI:** 10.1038/s41408-021-00445-z

**Published:** 2021-03-06

**Authors:** Alessandro Rambaldi, Alessandra Iurlo, Alessandro M. Vannucchi, Bruno Martino, Attilio Guarini, Marco Ruggeri, Nikolas von Bubnoff, Marianna De Muro, Mary Frances McMullin, Stefania Luciani, Vincenzo Martinelli, Axel Nogai, Vittorio Rosti, Alessandra Ricco, Paolo Bettica, Sara Manzoni, Silvia Di Tollo

**Affiliations:** 1 Department of Oncology and Hematology University of Milan, and Azienda SocioSanitaria Territoriale Papa Giovanni XXIII, Bergamo, Italy; 2 Hematology Division, Fondazione IRCCS Ca’ Granda Ospedale Maggiore Policlinico, Milan, Italy; 3 Center Research and Innovation of Myeloproliferative Neoplasms, AOU Careggi, University of Florence, Florence, Italy; 4 Grande Ospedale Metropolitano Bianchi-Melacrino-Morelli, Haematology Unit, Reggio Calabria, Italy; 5 Hematology Unit, IRCCS Istituto Tumori “Giovanni Paolo II”, Bari, Italy; 6 U.O. Haematology, San Bortolo Hospital, Vicenza, Italy; 7 Department of Haematology, Oncology and Stem Cell Transplantation, Medical Center, Faculty of Medicine, University of Freiburg, Freiburg, Germany; 8 Department of Haematology and Oncology, Medical Center, University of Schleswig-Holstein, Campus Lübeck, Lübeck, Germany; 9 Hematology and Stem Cells Transplantation Unit, Campus Bio-Medico, University Hospital, Rome, Italy; 10 Centre for Medical Education, Queen’s University Belfast, Belfast, United Kingdom; 11 Dipartimento Oncologia-Ematologia, U.O. Complessa Ematologia Clinica, Presidio Ospedaliero “Spirito Santo”- A.S.L. Azienda Sanitaria Locale, Pescara, Italy; 12 Dipartimento di Medicina Clinica e Chirurgia, Ematologia, Università degli Studi di Napoli Federico II, Naples, Italy; 13 Division of Hematology and Oncology at Campus Benjamin Franklin (CBF), Charité, Berlin, Germany; 14 Fondazione I.R.C.C.S. Policlinico San Matteo di Pavia, Centro per lo Studio e la Cura della Mielofibrosi, Laboratorio Biochimica, Biotecnologie e Diagnostica Avanzata, Pavia, Italy; 15 Azienda Ospedaliero-Universitaria Policlinico Consorziale di Bari, U. O. Ematologia con Trapianto – Ambulatorio, Bari, Italy; 16 Clinical R&D Department, Italfarmaco S.p.A, Cinisello Balsamo, Italy

**Keywords:** Drug development, Myeloproliferative disease

## Abstract

Polycythemia vera (PV) is a *BCR-ABL1*-negative myeloproliferative neoplasm (MPN) characterized by excessive proliferation of erythroid, myeloid, and megakaryocytic components in the bone marrow, mainly due to a Janus kinase 2 gene mutation (*JAK2*^V617F^). Givinostat, a histone-deacetylase inhibitor that selectively targets *JAK2*^V617F^ cell growth, has demonstrated good efficacy and safety in three phase 1/2 studies in patients with PV. This manuscript focuses on the 4-year mean (2.8 year median) follow-up of an open-label, long-term study that enrolled 51 patients with PV (out of a total of 54 with MPN) who received clinical benefit from givinostat in these previous studies or on compassionate use, and who continued to receive givinostat at the last effective and tolerated dose. The primary objectives are to determine givinostat’s long-term safety and tolerability, and efficacy evaluated by the investigators according to internationally recognized response criteria. During follow-up, only 10% of PV patients reported Grade 3 treatment-related adverse events (AEs), while none had Grade 4 or 5 treatment-related AEs. The overall response rate for the duration of follow-up was always greater than 80% in patients with PV. In conclusion, givinostat demonstrated a good safety and efficacy profile in patients with PV, data supporting long-term use in this population.

## Introduction

The *BCR-ABL1*-negative chronic myeloproliferative neoplasms (MPNs) are a group of hematological malignancies including polycythemia vera (PV), essential thrombocythemia (ET), and myelofibrosis (MF)^1^. PV is characterized by excessive production of progenitors and mature cells of the erythroid lineage with increased red cells, white blood cells, and platelets, whereas ET features hyperplasia of megakaryocytic lineage, and MF involves bone marrow fibrosis and granulocytic and megakaryocytic lineage expansion^1–3^. There is significant heterogeneity in symptom burden both within and between MPN subtypes, but all are characterized by vascular and hemorrhagic complications, and evolution to myelodysplasia, acute myeloid leukemia (AML), and/or fibrotic spent phase, with significant impact on patients’ quality of life and reduced survival^4^.

The National Comprehensive Cancer Network added MPNs to their guidelines in the autumn of 2016, with specific treatment guidelines for PV added in the summer of 2017^5^. In January 2018, the European LeukemiaNet (ELN) published revised management recommendations for MPNs^6^. According to these, current therapies aim to normalize hematocrit by use of phlebotomy and low-dose aspirin, if not contraindicated^6^. Hydroxyurea is used as first-line therapy when cytoreduction is necessary^7^, although toxicity can result in inadequate disease management^8^. Ruxolitinib is second-line therapy for patients who are refractory and/or intolerant to hydroxyurea;^5^ other treatments include busulfan, and non-pegylated and pegylated interferon (off-label)^7,9,10^, but use is limited by side effects and safety concerns. More recently, ropeginterferon α-2b was approved by the European Medicines Agency as first-line therapy for patients with PV without symptomatic splenomegaly when cytoreduction is necessary, having demonstrated non-inferiority to hydroxyurea in a Phase 3 study^11^. Hydroxyurea is also first-line therapy for patients with ET who need cytoreduction, with pegylated interferon and anagrelide used second-line or in selected cases (the very young or, in the case of pegylated interferon, during pregnancy)^6^. In MF, the only therapeutic approach to impact disease progression is allogeneic hematopoietic stem cell transplant, but this is associated with increased morbidity and mortality^12^, and although ruxolitinib has been approved in both the US and Europe, the authors of the ELN management recommendations consider its use justified only in specific settings^6^. Novel, targeted therapies are, therefore, needed for MPNs.

The genetic basis of MPNs has been extensively studied: up to 95% of patients with PV and over 50% of those with ET or MF have a mutation in the Janus kinase 2 (*JAK2*) gene, exon 14, in which valine is replaced with phenylalanine in position 617 (*JAK2*^V617F^), resulting in activation of biochemical pathways implicated in erythropoietin receptor signaling^13–19^. Givinostat is a histone-deacetylase inhibitor that selectively targets *JAK2*^V617F^ cell growth and reduces proliferation of hematopoietic cells^20^. The efficacy and safety of givinostat in MPNs has previously been evaluated in three open-label, multicenter, 24-week studies^21–23^. The first was a pilot study in patients with *JAK2*^V617F^-positive MPN who were either refractory to hydroxyurea, or were young and required cytoreductive therapy (in this population hydroxyurea was not recommended, but no other treatment was available in 2007 when the study started)^21^. In this trial 29 patients with MPN (12 with PV, one with ET, and 16 with MF) received givinostat monotherapy at a daily dose of 50–150 mg^21^. The second was a Phase 2 study that enrolled 44 patients with *JAK2*^V617F^-positive PV who were non-responders (NR) to the maximum tolerated dose (MTD) of hydroxyurea monotherapy for at least 3 months; in this study, givinostat was again administered at a daily dose of 50–150 mg, although in addition to the MTD of hydroxyurea^22^. The third was a two-part study that enrolled 48 patients with *JAK2*^V617F^-positive PV; Part 1 aimed to identify the MTD of givinostat in patients with PV, while the aim of Part 2 was to evaluate the efficacy and safety of givinostat’s MTD (i.e., 200 mg/day)^23^. Overall, in these three Phase 1/2 studies, givinostat (either alone or in combination with hydroxyurea) was effective, with a high response rate (50–80% depending on study and dose administered) and a good safety profile, with the majority of the observed adverse events (AEs) being mild or moderate in severity.

In the absence of any other effective drug, 78 of 121 (64.5%) patients with MPN (71 out of 104 [68.3%] patients with PV) who received clinical benefit from givinostat in these studies (described as the “core studies” in this manuscript) entered a named-patient compassionate use program, with 53 of the 78 (67.9%) patients with MPN subsequently entering the long-term study. Patients were also eligible for the long-term study if they had received compassionate use of givinostat without participating in one of the core studies—although in the event this applied only to one recruited patient. The results of the first interim analysis of this long-term study are summarized in this manuscript. At the cut-off date of 31 December 2018, the first patient to enter had completed 69 months (nearly 6 years) of follow-up in this long-term study, for a total of more than 11 years of givinostat treatment from the first intake in the core study. Although recruitment is open to patients with any MPN, at the cut-off date of this first interim analysis no patient with ET and only three with MF (with a maximum follow-up of 9 months in this long-term study) had been recruited. Given the limited data on these other populations, this manuscript therefore focuses on the 4-year mean follow-up results of the PV subset.

## Materials and methods

### Study design and patients

This is an ongoing, multicenter, open-label, single-arm, long-term study that aims to evaluate the safety, tolerability, and efficacy of givinostat (alone or in combination with hydroxyurea) in patients with MPN. The trial is registered in Clinicaltrials.gov (NCT01761968).

Eligible patients are *JAK2*^V617F^-positive, ≥18 years of age, with an established diagnosis of MPN according to the revised World Health Organization criteria, who had tolerated previous givinostat treatment and achieved a clinical benefit at the end of either three core studies^21–23^ and/or a compassionate use program, and provided written informed consent prior to any study-related procedure. The study was approved by the independent ethics committees at each institution, and was performed in accordance with the principles of the Declaration of Helsinki, and the International Conference on Harmonization notes for guidance on Good Clinical Practice (ICH/CPMP/135/95).

Patients continue to receive givinostat (alone or in combination with hydroxyurea if co-administered in the core study^22^) at the last tolerated dose and regimen at the end of the core study or compassionate use program. However, since the MTD of givinostat for chronic treatment for PV was identified after the end of the first two core studies^23^, patients entering from these two studies could have their givinostat dose escalated up to this MTD to potentially improve treatment response. Dose adjustments are allowed to optimize an individual patient’s response, while minimizing treatment-related AEs. Participation in the study may continue up to marketing authorization of givinostat for MPN in each country, although this was not achieved at the time of this interim analysis.

Consistent with clinical practice, patients attend investigational sites every three months, with additional visits when necessary—for example to monitor any AEs or unexpected conditions. Safety and tolerability are evaluated at these visits by the assessment of vital signs, blood chemistry, electrocardiogram, hematology, and Eastern Cooperative Oncology Group^24^ performance status. In addition, investigators assess clinical benefit based on internationally recognized response criteria, specifically the clinico-hematological ELN (see supplement)^24^ and EUMNET^25^ response criteria for patients with PV/ET and MF, respectively. Finally, during each visit, investigators evaluate the medical risk:benefit ratio for each patient, taking into account safety and tolerability, clinical benefit, and any alternative therapeutic options available at the time of the visit. In addition, during annual visits, blood samples are collected in order to evaluate disease-specific biomarkers.

### Study objectives

The primary objectives of this long-term study are to determine the long-term safety and tolerability of givinostat (in terms of AEs), and efficacy evaluated by investigators according to internationally recognized response criteria^24,25^. For patients with PV/ET, given the long-term nature of this study these criteria were revised, with the spleen evaluation not limited to imaging assessments (e.g., magnetic resonance imaging or computed tomography scans) but permitting assessment by palpation, consistent with clinical practice. The overall response rate included those who met the definitions of complete response (CR) or partial response (PR; see supplement for the response criteria for PV criteria). The type, incidence and severity of AEs was graded according to Common Terminology Criteria for AEs (CTCAE) version 4.03.

As an exploratory endpoint, *JAK2*^V617F^ allele burden is being evaluated at the end of each year using quantitative real-time polymerase chain reaction (qRT-PCR). Several exploratory endpoints are being assessed in order to better study givinostat’s long-term efficacy profile, although results are not included in this manuscript. These include treatment effect on each response parameter, as evaluated by investigators according to internationally recognized response criteria^24,25^ (for instance, for patients with PV, response in terms of white blood cells, platelets, hematocrit without phlebotomy, disease-related symptoms, and spleen). In addition, other potential biomarkers predictive of clinical benefit of givinostat (e.g., pharmacodynamic biomarkers) are being evaluated.

### Sample size and statistical methods

Due to the exploratory nature of the study, it was not formally powered. All safety data are summarized descriptively, with the frequency and percentage of patients in each response category reported annually.

To take a conservative approach, all patients who withdrew from the study due to disease progression and/or treatment-related AEs (regardless of the relationship between AE onset and time of withdrawal from the study) were considered “non-responders”; while patients who withdrew due to safety reasons other than due to treatment-related AEs (e.g., non-related AEs, investigator’s decision, poor compliance) and/or who withdrew consent were captured in the responder analysis as “other” reason for drop-out. Data are presented for “real baseline” (which are from assessments conducted prior to a patient’s first givinostat administration on entry to the core study), “end of core” (on completion of the core study), and “baseline” (prior to commencing the long-term study; of note, all patients were already receiving givinostat at this timepoint).

Safety analyses were conducted on the safety population, which included all recruited patients who received at least one dose of study drug in the long-term study. Efficacy analyses were conducted on the intent-to-treat (ITT) population, which included all patients in the safety population from whom at least one post-baseline efficacy measurement was obtained in the long-term study. A series of post-hoc analyses were also performed with patients subgrouped by concomitant hydroxyurea use.

## Results

### Participants

This manuscript reports the results from the first interim analysis (data cut-off date: 31 December 2018) of this ongoing, long-term study. The first patient received their first givinostat dose in the long-term study on 28 March 2013, with the last patient included in this analysis receiving their first dose on 25 September 2017.

A total of 54 patients with MPNs have been enrolled by 15 sites across Europe (Italy, UK, France, and Germany). Although recruitment is open to all patients with MPN, at the cut-off date no patients with ET, and only three with MF (one with primary MF, one with post-PV MF, and one with post-ET MF) were recruited, with a maximum follow-up of 9 months in this long-term study. Therefore, due to limited data on the other two populations (i.e., ET and MF), this manuscript focuses on the follow-up results of the PV subset.

One of the 51 patients with PV failed screening (prolonged QTc); all the remaining patients received at least one dose of givinostat in this long-term study (range: 50 mg once a day to 100 mg twice a day). In this first interim analysis, the safety and ITT populations therefore included 50 patients. Table 1 summarizes the demographics and disease characteristics of the PV subset at real baseline. The mean time since diagnosis on entry to the long-term study was more than 7 years. Fifteen of these patients received concomitant hydroxyurea during the long-term study; the baseline demographics and disease characteristics of this subgroup were similar to those of the patients who did not receive hydroxyurea (Supplemental Table 1). The mean time since diagnosis on entry to the long-term study was more than 5 years in the subgroup who did not receive concomitant hydroxyurea, and more than 11 years in those who did receive concomitant hydroxyurea. During the long-term study, investigators discontinued concomitant hydroxyurea in ten of these patients (66.7%), and the dosage of hydroxyurea in this subgroup consequently reduced from a median of 500 mg per day (range: 286 to 1750 mg; mean: 688 mg) at real baseline to 0 mg per day (range: 0 to 1000 mg; mean: 192 mg) at the cut-off date of the interim analysis.

**Table 1:** Demographics and disease characteristics for the treated PV subset at real baseline.

	PV subset (*N* = 50)
Age in years, median (range)	59.0 (42 to 80)
Age group, number (%)	
<60 years	25 (50.0)
≥60 years	25 (50.0)
Sex, number (%)	
Male	31 (62.0)
Female	19 (38.0)
Race, white, number (%)	50 (100)
Time since diagnosis in years, ^a^mean ± SD	7.2 ± 5.70
Controlled hypertension, number (%)	29 (58.0)
Hematology, median (range)	
Hemoglobin, g/l	136 (118 to 166)
Hematocrit, %	45.0 (40.9 to 50.7)
Platelets, 10^9^/l	698 (264 to 1459)
White blood cells, 10^9^/l	13.44 (3.71 to 46.48)
Patients requiring phlebotomy, number (%)	47 (94.0)
*JAK2*^V617F-^positivity (%)	50 (100)
*JAK2*^V617F^ allele burden, %, median (range)	59.75 (25.0 to 94.2)
Prior therapy for PV, number (%) [maximum daily dose]	
Antiplatelet treatments	26 (52.0)
Acetylsalicylic acid	24 (48.0) [100 mg]
Ticlopidine	2 (4.0) [250 mg]
Clopidogrel	1 (2.0) [75 mg]
Cytoreductive treatments	30 (60.0)
Hydroxyurea	24 (48.0) [1.5 g]
Interferon	4 (8.0) [1.5 × 10^6^ U]
Busulfan	2 (4.0) [4 mg]
Number of different prior PV therapies, number (%)	
0	11 (22.0)
1	23 (46.0)
2	12 (24.0)
3	4 (8.0)

*PV* polycythemia vera, *JAK* Janus kinase.

^a^At time of entry to the long-term study.

At the time of the cut-off for the interim analysis, mean exposure to givinostat was 4.2 years from the first intake (median: 2.8 years; range: 3 months to 11 years); 31 out of 50 patients with PV (62%) were still receiving treatment. Ten patients withdrew from the study due to AEs, six due to treatment-related AEs although only one with AEs above CTCAE Grade 2 (diarrhea, resolving in 14 days without treatment). One of the patients who withdrew due to a related AE (Grade 1 anemia) died during the follow-up period (1 month after discontinuing givinostat) due to a serious non-related AE (worsening of thromboembolism in the left leg).

Four patients withdrew due to disease progression (one to AML, and three to post-PV MF), one due to physician’s decision (subsequently confirmed to have disease progression to post-PV MF), and one due to lack of response (subsequently confirmed to be progression to post-PV MF)—so a total of five patients evolved to post-PV MF and only one to AML. Two patients withdrew consent, and one died, with the event judged not drug-related and without other cause (i.e., natural death).

### Safety

During the long-term study, 48 of the 50 patients (96.0%) experienced at least one AE, although most of the AEs reported over this period were Grade <3 (393/438 events; 89.7%). Furthermore, the majority of AEs considered related to treatment were Grade <3 (111/118 events; 94.1%), with only five patients (10.0%) experiencing Grade 3 events; no treatment-related Grade 4 or Grade 5 AEs occurred (Table 2). Three patients (6.0%) experienced drug-related QTc prolongation during treatment with givinostat, but only one patient (2.0%) had a Grade 3 event, which resulted in treatment discontinuation. QTc prolongation is a well-known side effect of givinostat, as are thrombocytopenia and gastrointestinal AEs. Thirteen patients (26.0%) experienced serious AEs during the long-term study (Supplemental Table 2), only one of whom (2.0%) had an AE that was recorded as study related (Grade 2 multiple sclerosis) due to the sponsor’s standard policies, although the event was judged by investigator as relationship to study “unknown”. Results were broadly similar when patients were subgrouped by concomitant hydroxyurea use (Supplemental Table 3). However, all patients who received concomitant hydroxyurea experienced at least one AE, a higher proportion of patients in this subgroup experienced serious AEs, and the Grade 3 treatment-related AEs of thrombocytopenia and asthenia were only experienced in this subgroup.

**Table 2:** Patients with study drug-related AEs during the long-term study, overall and by system organ class and preferred term (including only preferred terms reported by one or more patient with Grade >2 events).

System organ class	Grade 3		Any grade	
Preferred term	*N*	%	*N*	%
**Patients with any treatment-related AE**	5	10.0	32	64.0
Blood and lymphatic system disorders	1	2.0	12	24.0
Thrombocytopenia	1	2.0	7	14.0
Cardiac disorders	0^a^	0	1	2.0
Eye disorders	0^a^	0	1	2.0
Gastrointestinal disorders	1	2.0	16	32.0
Diarrhea	1	2.0	12	24.0
General disorders and administration site conditions	1	2.0	3	6.0
Asthenia	1	2.0	3	6.0
Infections and infestations	0^a^	0	2	4.0
Investigations	1	2.0	9	18.0
Electrocardiogram QT prolonged	1	2.0	3	6.0
Metabolism and nutrition disorders	0^a^	0	1	2.0
Nervous system disorders	0^a^	0	4	8.0
Psychiatric disorders	0^a^	0	2	4.0
Skin and subcutaneous tissue disorders	0^a^	0	6	12.0
Vascular disorders	1	2.0	1	2.0
Hypertension	1	2.0	1	2.0

Grades are based on CTCAE Version 4.03, where Grade 1 are mild events, Grade 2 are moderate, Grade 3 are severe, Grade 4 are life-threatening, and Grade 5 events result in death. “Any grade” means any Grade 1, 2, 3, 4, or 5 events. There were no Grade 4 or 5 treatment-related events.

*AE* adverse event, *CTCAE* common terminology criteria for AEs.

^a^There were no Grade 3 events for these system organ classes.

### Efficacy

**Fig. 1:**
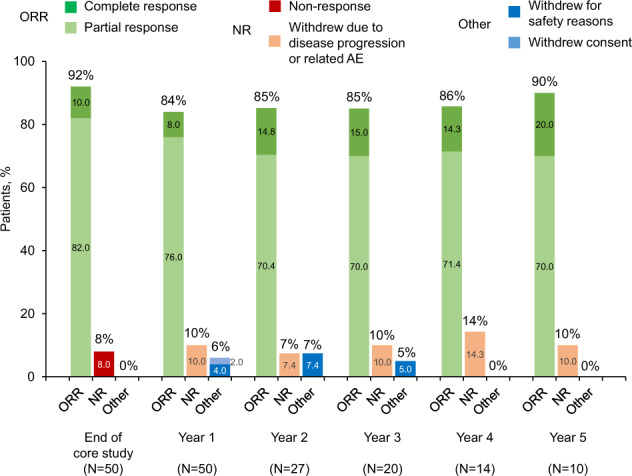
Therapeutic response evaluation according to revised clinico-hematological ELN criteria (ITT population). Note that as this is an ongoing study, patients currently in the study have received varying durations of therapy. At the time of these analyses, 31 patients were still in the study. Percentages are calculated from the number of patients with data available at each timepoint (sum of the patients attending the stated visit and patients who withdrew from the study between visits due to disease progression or related AE, withdrew for safety reasons or withdrew consent). ORR overall response rate (total of partial and complete response), NR non-responder, AE adverse event.

**Fig. 2:**
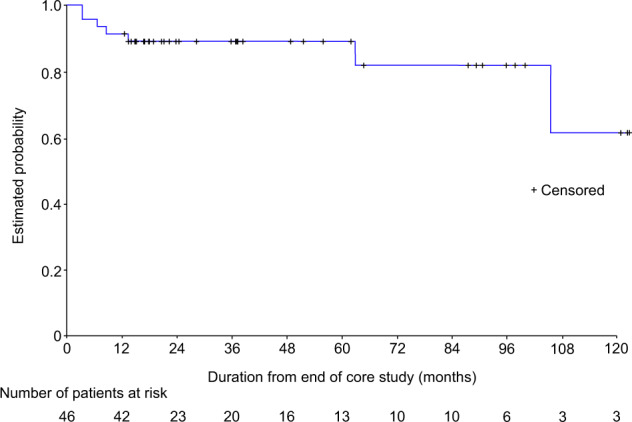
Time to treatment failure of responders (according to revised clinico-hematological ELN criteria) (ITT Population). The X axis is the duration from the end of the core study (in months), and not visits. Note that as this is an ongoing study, patients currently in the study have received varying durations of therapy. At the time of these analyses, 31 patients were still in the study.

More than 80% of patients had a PR or CR, maintained for the duration of follow-up (Figs. 1, 2, respectively). Of note, four patients (8.0%) met the definition of NR on entry to the long-term study, but were enrolled based on the fact that they were receiving clinical benefit from compassionate use of givinostat in terms of disease-related symptoms and/or hematological parameters, with no other effective treatment available. When subgrouped by concomitant hydroxyurea use, overall response at the end of the core study was higher in the givinostat monotherapy subgroup than in the concomitant hydroxyurea subgroup (Supplemental Fig. 1). Importantly, a reduction in mean *JAK2*^*V617F*^ allele burden was observed at most of the annual visits (Table 3).

**Table 3:** JAK2^V617F^ allele burden (ITT population).

Timepoint	Absolute *JAK2*^V617F^ allele burden, %
Real baseline (*N* = 50)	60.1 ± 18.94
Year 1 (*N* = 42)	48.5 ± 20.01
Year 2 (*N* = 19)	50.6 ± 21.49
Year 3 (*N* = 11)	58.9 ± 26.86
Year 4 (*N* = 10)	47.2 ± 24.71
Year 5 (*N* = 9)	42.1 ± 14.05

Note that as this is an ongoing study, patients currently in the study have received varying durations of therapy. At the time of these analyses, 31 patients were still in the study. Data are mean ± standard deviation.

*JAK* Janus kinase.

## Discussion

This is the first study to evaluate the long-term safety and efficacy profile of givinostat in MPNs. This manuscript reports the first interim analysis results from a mean follow-up of 4 years, of 51 patients with PV (out of a total of 54 with MPN), who had received clinical benefit from givinostat in three previous Phase 1/2 studies and/or on compassionate use, and who continued to receive givinostat at the last effective and tolerated dose. Notably, the majority of patients with MPN continued treatment with givinostat at the end of the three *core* studies, as they were receiving a relevant clinical benefit from the treatment. Givinostat was confirmed to have a good overall safety and tolerability profile: although nearly all patients experienced at least one AE during the long-term study, the majority were Grade <3 and were not considered related to treatment. The overall AE profile was consistent with that observed in previous studies, with no new safety concerns observed^21–23,26–32^. The majority of patients with PV were still receiving givinostat therapy at the time of this interim analysis, some of whom had been receiving chronic givinostat therapy for 11 years. Only six patients with PV reported disease progression (one to AML and five to post-PV MF), representing a rate of disease progression of 2.8 patients per year; the range of reported frequencies for post-PV AML are 2.3–14.4% at 10 years after diagnosis, and for post-PV MF are 4.9–6.0% at 10 years^33^.

In term of response rate, the overall response rate for the duration of follow-up was always greater than 80% in patients with PV in this long-term study. A reduction in mean *JAK2*^V617F^ allele burden was observed at most of the annual visits. In the post-hoc analyses by concomitant hydroxyurea use, although the baseline characteristics of the two sub-groups were similar, the AE profile was slightly better in patients who did not received concomitant hydroxyurea (with a lower overall occurrence of severe AEs), whereas interestingly, the overall response was higher in the givinostat monotherapy subgroup.

The study does have some limitations. As an ongoing long-term study, and with the lengthy recruitment period, patients are at various stages of treatment, with few having full 4 and 5-year data. In addition, almost all those recruited had PV. This limits the conclusions that can be drawn to patients with PV. Further, the single-arm, non-comparative nature of the study means that no conclusion can be drawn on the relative efficacy profile of givinostat to other treatments for PV. Finally, only patients who had previously achieved clinical benefit from givinostat were recruited. However, these are the patients who would receive long-term therapy with givinostat, again making the results potentially generalizable.

In conclusion, givinostat was confirmed to have a good safety and efficacy profile in patients with PV, with data supporting long-term use in this population.

## Supplementary information

Supplementary material
