# Supplementary material for: Long-term safety and efficacy of givinostat in polycythemia vera: 4-year mean follow up of three phase 1/2 studies and a compassionate use program
Source: Blood Cancer J. 2021 Mar 6;11(3):53. doi: 10.1038/s41408-021-00445-z (PMC7936975; doi:10.1038/s41408-021-00445-z)
Supplement: Supplementary file 1 — Supplementary material [file 41408_2021_445_MOESM1_ESM.pdf]

Long-term safety and efficacy of givinostat in polycythemia vera: 4-year mean follow up of three phase 1/2 studies and a compassionate use program

Authors

Alessandro Rambaldi, Alessandra Iurlo, Alessandro M. Vannucchi, Bruno Martino, Attilio Guarini, Marco Ruggeri, Nikolas von Bubnoff, Marianna De Muro, Mary Frances McMullin, Stefania Luciani, Vincenzo Martinelli, Axel Nogai, Vittorio Rosti, Alessandra Ricco, Paolo Bettica, Sara Manzoni, and Silvia Di Tollo

Supplement

Methods

Response criteria for PV (from the revised clinico-hematological ELN response criteria):

- **Complete response:**
  1. Hematocrit < 45% without phlebotomy, and
  2. Platelet count ≤ 400 x 10^9/l, and
  3. White blood cell count ≤ 10 x 10^9/l, and
  4. Normal spleen size, and
  5. No disease related systemic symptoms (i.e., microvascular disturbances, pruritus, headache).
- **Partial response:**

  Patients who do not fulfil the criteria for complete response and
  1. Hematocrit < 45% without phlebotomy, or
  2. Response in three or more of the other criteria.
- **No response:** Any response that does not satisfy partial response.

Results

Supplemental Table 1. Demographics and disease characteristics for the treated PV subset at real baseline, with patients sub-grouped by hydroxyurea use at real baseline

	**No concomitant hydroxyurea (N=35)**	**Concomitant hydroxyurea (N=15)**
Age in years, median (range)	58.0 (42 to 80)	60.0 (42 to 76)
Age group, number (%)		
< 60 years	18 (51.4)	7 (46.7)
≥ 60 years	17 (48.6)	8 (53.3)
Sex, number (%)		
Male	22 (62.9)	9 (60.0)
Female	13 (37.1)	6 (40.0)
Race, white, number (%)	35 (100)	15 (100)
Time since diagnosis in years,* mean ± SD	5.5±4.51	11.3±6.20
Controlled hypertension, number (%)	17 (48.6)	12 (80.0)
Hematology, median (range)		
Hemoglobin, g/l	132 (118 to 163)	141 (126 to 166)
Hematocrit, %	45.0 (40.9 to 50.7)	45.0 (41.5 to 50.5)
Platelets, 10^9/l	704 (285 to 1332)	489 (264 to 1459)
White blood cells, 10^9/l	15.11 (4.50 to 46.48)	9.00 (3.71 to 23.10)
Patients requiring phlebotomy, number (%)	34 (97.1)	13 (86.7)
JAK2^{V617F}-positivity (%)	35 (100)	15 (100)
JAK2^{V617F} allele burden, %, median (range)	66.00 (26.0 to 94.2)	47.00 (25.0 to 71.1)
Prior therapy for PV, number (%) [maximum daily dose]		
Antiplatelet treatments	24 (68.6)	2 (13.3)
Acetylsalicylic acid	23 (65.7) [100 mg]	1 (6.7)
Ticlopidine	2 (5.7) [250 mg]	0
Clopidogrel	1 (2.9) [75 mg]	0
Cytoreductive treatments	16 (45.7)	9 (60.0)
Hydroxyurea	16 (45.7) [1.5 g]	8 (53.3) [1.5 g]
Interferon	3 (8.6) [1.5x10^6 U]	1 (6.7)
Busulfan	1 (2.9) [4 mg]	1 (6.7) [2 mg]

	**No concomitant hydroxyurea (N=35)**	**Concomitant hydroxyurea (N=15)**
Number of different therapies, number (%)		
0	5 (14.3)	6 (40.0)
1	18 (51.4)	6 (40.0)
2	10 (28.6)	3 (20.0)
3	2 (5.7)	0

PV, polycythemia vera; JAK, Janus kinase. *At time of entry to the long-term study.

Supplemental Table 2. Patients with serious treatment-emergent AEs, overall and by system organ class and preferred term (including only preferred terms reported by one or more patient with Grade 3 events)

System organ class<br>Preferred term	Grade 3		Grade 4		Grade 5		Any grade	
	N	%	N	%	N	%	N	%
**Patients with at least one treatment-related serious AE**	6	12.0	1	2.0	2	4.0	13	26.0
Cardiac disorders	1	2.0	0	0	0	0	1	2.0
Cardiac failure congestive	1	2.0	0	0	0	0	1	2.0
Gastrointestinal disorders	1	2.0	0	0	0	0	1	2.0
Anal fissure	1	2.0	0	0	0	0	1	2.0
General disorders and administration site conditions	0	0	0	0	1	2.0	1	2.0
Sudden death	0	0	0	0	1	2.0	1	2.0
Hepatobiliary disorders	1	2.0	0	0	0	0	1	2.0
Cholecystitis acute	1	2.0	0	0	0	0	1	2.0
Infections and infestations	1	2.0	1	2.0	0	0	4	8.0
Pneumonia	1	2.0	0	0	0	0	2	4.0
Sepsis	0	0	1	2.0	0	0	1	2.0
Injury, poisoning and procedural complications	0	0	0	0	1	2.0	1	2.0
Post procedural complication	0	0	0	0	1	2.0	1	2.0
Investigations	1	2.0	0	0	0	0	1	2.0
Blood creatinine increased	1	2.0	0	0	0	0	1	2.0
Neoplasms benign, malignant and unspecified (incl cysts and polyps)	1	2.0	0	0	0	0	2	4.0
Cancer pain	1	2.0	0	0	0	0	1	2.0
Nervous system disorders	0*	0	0*	0	0*	0	3	6.0
Renal and urinary disorders	2	4.0	0	0	0	0	2	4.0
Acute kidney injury	1	2.0	0	0	0	0	1	2.0
Nephrolithiasis	1	2.0	0	0	0	0	1	2.0
Vascular disorders	2	4.0	0	0	0	0	4	8.0

System organ class<br>Preferred term	Grade 3		Grade 4		Grade 5		Any grade	
	N	%	N	%	N	%	N	%
Embolism	1	2.0	0	0	0	0	1	2.0
Peripheral arterial occlusive disease	1	2.0	0	0	0	0	1	2.0

Grades are based on the CTCAE Version 4.03, where Grade 1 are mild events, Grade 2 are moderate, Grade 3 are severe, Grade 4 are life-threatening, and Grade 5 events result in death. *There were no Grade 3, 4 or 5 events for these system organ classes. AE, adverse event.

Supplemental Table 3. Overall experience of AEs, with patients sub-grouped by concomitant hydroxyurea use

	**No concomitant hydroxyurea (N=35)**	**Concomitant hydroxyurea (N=15)**
At least one AE	33 (94.3)	15 (100)
At least one treatment-related AE	24 (68.6)	8 (53.3)
At least one treatment-related CTCAE Grade 3 AE*	3 (8.6)	2 (13.3)
Thrombocytopenia	0	1 (6.7)
Diarrhea	1 (2.9)	0
Asthenia	0	1 (6.7)
Electrocardiogram QT prolonged	1 (2.9)	0
Hypertension	1 (2.9)	0
At least one serious AE	6 (17.1)	7 (46.7)
At least one treatment-related serious AE	1 (2.9)	0

*Grades are based on the CTCAE Version 4.03, where Grade 3 are severe. There were no Grade 4 or 5 treatment-related events. AE, adverse event. CTCAE, Common Terminology Criteria for AEs.

Supplemental Figure 1. Therapeutic response evaluation according to revised clinico-hematological ELN criteria with patients sub-grouped by concomitant hydroxyurea use

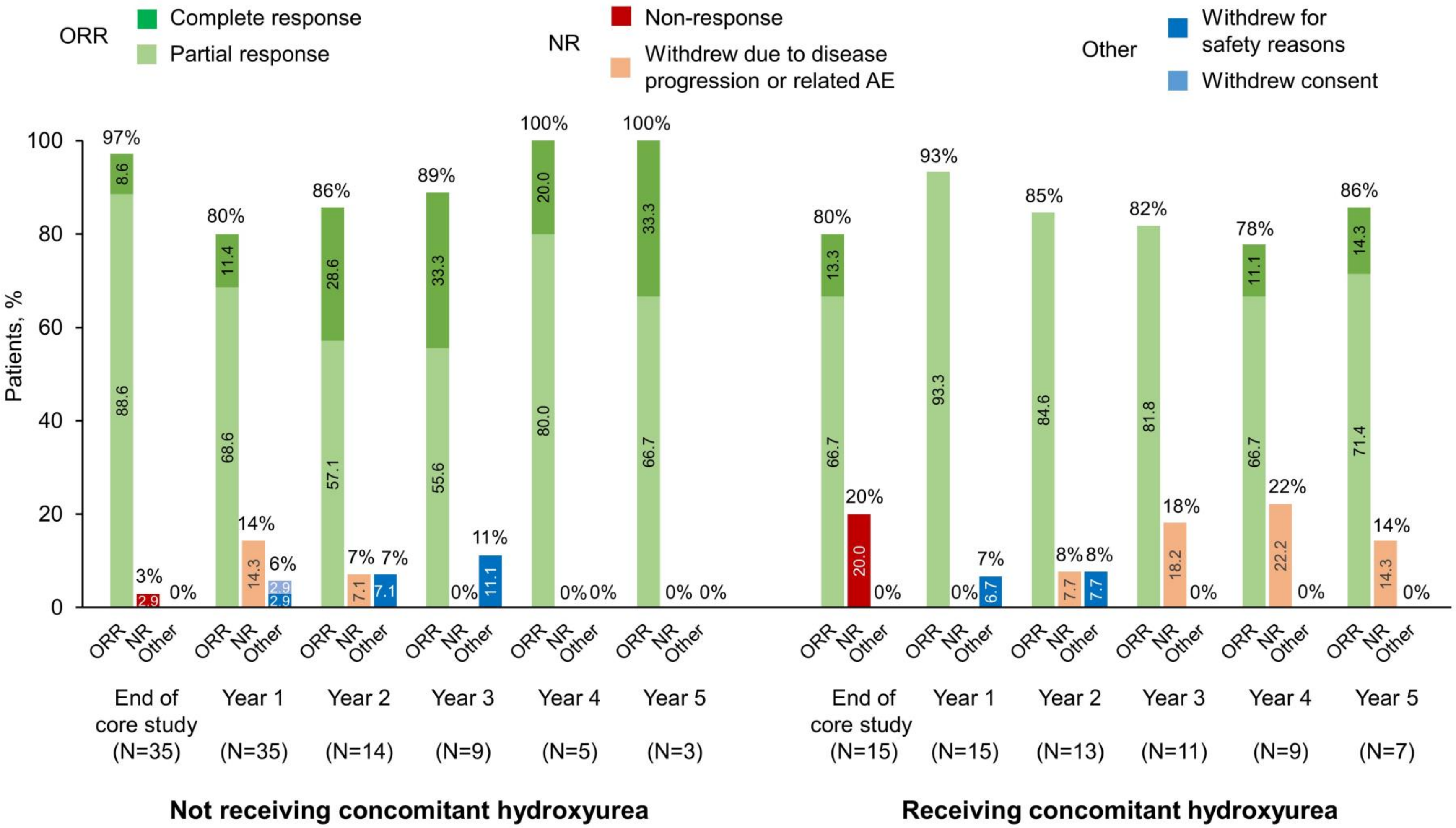

Note that as this is an ongoing study, patients currently in the study have received varying durations of therapy. Percentages are calculated from the number of patients with data available at each timepoint (sum of the patients attending the stated visit and patients who withdrew from the study between visits due to disease progression or related AE, withdrew for safety reasons or withdrew consent). ORR, overall response rate (total of partial and complete response); NR, non-responder; AE, adverse event.
